# Development and validation of an instrument for the evaluation of HIV care in Primary Health Care

**DOI:** 10.1590/0034-7167-2022-0247

**Published:** 2023-01-30

**Authors:** Clarissa Mourão Pinho, Morgana Cristina Leôncio de Lima, Mônica Alice Santos Silva, Cynthia Angélica Ramos de Oliveira Dourado, Regina Célia de Oliveira, Jael Maria de Aquino, Erika Simone Galvão Pinto, Maria Sandra Andrade

**Affiliations:** I Universidade de Pernambuco. Recife, Pernambuco, Brazil; II Universidade Federal do Rio Grande do Norte. Natal, Rio Grande do Norte, Brazil

**Keywords:** HIV, Acquired Immunodeficiency Syndrome, Primary Health Care, Validation Study, Health Evaluation, VIH, Síndrome de Inmunodeficiencia Adquirido, Atención Primaria de Salud, Estudio de Validación, Evaluación en Salud, HIV, Síndrome da Imunodeficiência Adquirida, Atenção Primária à, Saúde, Estudo de validação, Avaliação em saúde

## Abstract

**Objective:**

To develop and validate an instrument to evaluate the decentralization process of care for People Living with HIV in Primary Health Care.

**Method:**

Methodological study, developed in four stages: elaboration of the logical model based on the triad *Structure-Process-Outcomes*; development of the instrument; content validation by expert judges and technical reviewers; and semantic validation. Online questionnaires were used, and the Kappa index was used for analysis.

**Results:**

The instrument with 68 items and 8 factors was submitted to validation by expert judges who recommended the exclusion of 3 items and the alteration of 2 factors. In the validation by technical reviewers, 2 items were excluded and 6 factors were highlighted; the agreement index was ≥0.75. In the semantic validation, 87.3% of the judges answered “totally agree” for the items presented.

**Conclusion:**

The instrument is validated for its content, has 63 items and has the potential to assess the care provided for people living with HIV in Primary Health Care.

## RESUMO

**Objetivo:**

construir e validar um instrumento de avaliação do processo de descentralização do atendimento às pessoas vivendo com HIV para Atenção Primária à Saúde.

**Métodos:**

estudo metodológico, desenvolvido em quatro etapas: elaboração do modelo lógico baseado na tríade *Estrutura-Processo-Resultados*; construção do instrumento; validação de conteúdo por juízes especialistas e revisores técnicos; e validação semântica. Utilizaram-se questionários on-line e, para análise, o índice de Kappa.

**Resultados:**

o instrumento, com 68 itens e 8 fatores, foi submetido à validação por juízes especialistas que recomendaram a exclusão de 3 itens e mudança de 2 fatores. Na validação por revisores técnicos, foram excluídos 2 itens e pontou-se 6 fatores, o índice de concordância foi ≥0,75. Na validação semântica 87,3% responderam “concordo totalmente” para os itens apresentados.

**Conclusões:**

O instrumento encontra-se validado quanto ao conteúdo, possui 63 itens e potencial para avaliação do atendimento às pessoas vivendo com HIV na Atenção Primária à Saúde.

## RESUMEN

**Objetivo:**

Construir y validar un instrumento para la evaluación del proceso de descentralización de la atención a personas conviviendo con VIH en la Atención Primaria de Salud.

**Métodos:**

Es un estudio metodológico desarrollado en cuatro etapas: elaboración del modelo lógico basado en la tríada *Estructura-Proceso-Resultados*; construcción del instrumento; validación del contenido por jueces especialistas y revisores técnicos; y validación semántica. Se utilizaron cuestionarios en línea y el índice de Kappa para el análisis.

**Resultados:**

El instrumento con 68 ítems y 8 factores fue sometido a validación por jueces expertos que recomendaron la exclusión de 3 ítems y el cambio de 2 factores. En la validación por parte de los revisores técnicos, se excluyeron 2 ítems y se puntuaron 6 factores; el índice de acuerdo fue ≥0,75. En la validación semántica, el 87,3% respondió “totalmente de acuerdo” a los ítems presentados.

**Conclusiones:**

El instrumento se encuentra validado cuanto al contenido, posee 63 ítems y potencial para la evaluación de la atención de personas conviviendo con VIH en la Atención Primaria de Salud.

## INTRODUCTION

In 2014, the Ministry of Health proposed a change in the care model for people living with HIV (PLHIV), considering Primary Health Care (PHC) as the health system gateway and main provider of care. The proposed alteration of the care model provides increased accessibility to health services for these users^(1)^. In Brazil, it is evident that this care process is still in the beginning. It requires effort, discussion and evaluations to assess the challenges and potentials of this health care model, which is an innovation in the care for PLHIV^(2-3)^.

The care process for PLHIV in PHC is already well established in other countries, especially in low-income African countries, where there is a high prevalence of HIV infection^(4-7)^. In Brazilian cities, it is possible to observe experiences of care decentralization for PLHIV in PHC in different stages of incorporation. It is a model that is currently being implemented, which can allow expanded access to health services for PLHIV^(8-10)^.

This expansion of access can increase testing, diagnosis and treatment rates, as well as HIV prevention programs with pre-exposure (PrEP) and post-exposure (PEP) prophylaxis^(8-10)^.In addition, it can enhance the bond between professionals and patients and optimize health education and treatment adherence actions, allowing the achievement of HIV infection control goals. However, it is clear that, to solidify this process and consolidate the success of the reorganization of health services, it is still necessary to overcome obstacles in technical, political, organizational and healthcare practice areas^(10-12)^.

Therefore, for the consolidation of this care model, there must be greater investment in the structure of Primary Care Centers, materials and resources, professional qualification and matrix support^(2-3)^. In this perspective, the evaluation of health services is of great importance for the consolidation of new care models and can contribute to identifying problems, making it possible to propose solutions to use resources more efficiently and effectively and to reorganize health care practices in political, economic, social and professional contexts^(13)^.

Health assessment instruments that allow measuring HIV care in PHC are still incipient compared to those that indicate the challenges and potentials of the care model^(2-6,9-13)^. It is worth mentioning that there is a national study that carried out the construction and validation of an instrument to evaluate the actions developed by health professionals in PHC to manage HIV/AIDS^(14)^. Thus, instruments that allow the evaluation of health services in which the decentralization process is already installed and that can assist in the structuring of health care centers that have not yet adhered to the recommendations can contribute to the consolidation of this healthcare model, considering the specificities and complexities of the consolidation of a new health care model.

## OBJECTIVES

To develop and validate an instrument to evaluate the decentralization process of care for People Living with HIV in Primary Health Care.

## METHODS

### Ethical aspects

The ethical precepts of Resolution 466/2012 were respected. The research project was approved by the Research Ethics Committee of the Hospital Universitário Oswaldo Cruz (HUOC)/Pronto Socorro Cardiológico Universitário de Pernambuco of Pernambuco - Prof. Luiz Tavares (PROCAPE).

### Study design, period and setting

This is a methodological study developed in four stages: elaboration of the logical model for the construction of the instrument to evaluate the process of decentralization of care for PLHIV in PHC; construction of the instrument; content and face validation by expert judges and technical reviewers; and semantic validation.

The triad Structure-Process-Outcomes of Avenis Donabedian^(15)^ was used as a theoretical framework for the elaboration of the logical model. The clinical protocol and the therapeutic guidelines for the management of HIV infection in adults^(16)^ and the HIV/AIDS kit for Primary Health Care launched by the Ministry of Health in 2017^(17-22)^ were used as references for the construction of the health assessment instrument. The construction and validation of the instrument occurred from January 2020 to March 2021, in the city of Recife, in the state of Pernambuco, Brazil.

### Population or sample; inclusion and exclusion criteria

A total of 10 expert judges were invited for the content validation process, of whom 9 agreed to participate. The following eligibility criteria were used: a) having a degree in a health-related course; b) having at least a stricto sensu or lato sensu specialization in collective health, public health, family health, infectiology or epidemiology; c) having at least 1 year of experience in the area of HIV/AIDS and sexually transmitted infections (STIs).

At this stage, in addition to the evaluation by the expert judges, the instrument was evaluated by technical reviewers. Of the 4 technical reviewers that were invited, 3 agreed to evaluate the instrument. The inclusion criteria for the technical reviewers were: a) being a health professional; b) having higher education; c) having at least 5 years of experience in the area of management or coordination of the STI/HIV/Aids Program.

Semantic validation was performed by 55 students. The following selection criteria were used: a) being in an undergraduate course in medicine or nursing; b) being ≥18 years of age; c) having attended classes on one of the subjects: primary health care, collective health, family health, infectious and parasitic diseases. Those who were in other courses in the health area were excluded. The inclusion of medical and nursing students is justified, as these students have theoretical and practical classes in the subjects of primary health care, collective health and family health in the first period of the undergraduate course.

### Study protocol

After the elaboration of the logical model^(15)^, the construction of the instrument to evaluate the process of decentralization of care for PLHIV in PHC began. The instrument was constructed by 4 researchers, all nurses. At this stage, the instrument was created with 68 items, distributed in 8 factors: Physical resources; Surveillance and notification; Diagnostic and routine tests; Treatment; Health prevention; Continuing Education; Health education; and Health Care Network. The items were evaluated using a *Likert*-type scale.

For the stage of content validation, which was performed by 9 expert judges, data was collected through an online Google form. The evaluators were given 30 days, counted from the day the document was sent, to accept the Informed Consent Form (TCLE) and answer the instrument. A key informant who worked in the STI/HIV/Aids Program of the Pernambuco State Health Department indicated the first judge, and the others were selected through the “snowball” strategy, which consists of appointing a new judge according to the indication of the previous one. When evaluating the content, the expert judges were asked to sign the option that best fitted the item. In addition, there was an “other” option, for the judges to suggest a new factor, and “observations” for them to give suggestions about the relevance, clarity, understanding, and reformulation of the item or recommend its exclusion. In this step, the alteration of two factors and the exclusion of 3 items were suggested. Thus, in the content evaluation by the expert judges, the instrument had 65 items and 7 factors.

After completing this step, the instrument was submitted to 3 technical reviewers. Data was collected electronically from September to November 2020. The technical reviewers suggested the exclusion of 2 items and 1 factor. Thus, in the evaluation by the technical reviewers, the instrument had 63 items and 6 factors. After the technical review, the instrument was proofread in Portuguese.

The semantic validation stage was conducted with the participation of 55 students from the University of Pernambuco, of which 21 were medical students and 34 were nursing students. Research participants received the invitation electronically. This stage occurred from December 2020 to March 2021. For the semantic validation, the students were asked to evaluate each item on verbal comprehension and select one of the options on a Likert-type scale, namely: 1) I don’t understand any of it; 2) I do not understand part of it; 3) I understand part of it; 4) I understand; 5) I understand it completely.

### Results and statistical analysis

For the analysis of the Instrument called “Evaluation of the decentralization process of care for People Living with HIV in Primary Health Care”, the standards presented by the American Educational Research Association (AERA), American Psychological Association (APA) and National Council on Measurement in Education (NCME)^(23)^ were followed for validation. Its precepts indicate that, for an instrument to be valid in its content, it is necessary to carry out some steps for validation. The stages of content validation by expert judges and/or technical reviewers and semantic validation were applied in this study.

Data collected through a form made available on Google forms allowed the elaboration of a database in an electronic spreadsheet on Microsoft Excel. Frequency analysis and measures of central tendency and dispersion were performed to characterize the sample and the Kappa coefficient (reference value ≥0.75)^(24-25)^ was calculated during the stages of content validation by expert judges, technical reviewers and semantic validation. The SPSS® software (Statistical Package for Social Science) version 26.0 was used for the statistical analysis.

## RESULTS

**Figure 1:**
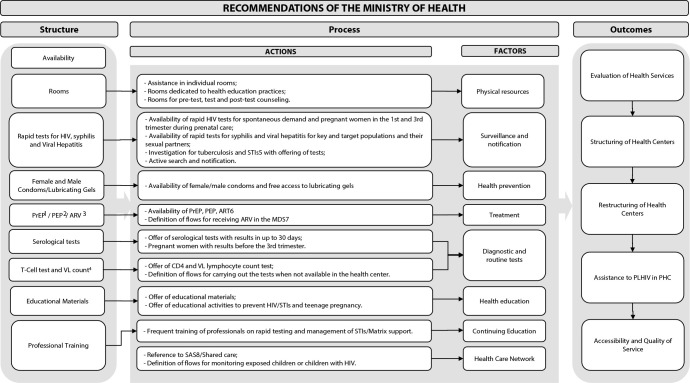
Logical model used in the development of the instrument for assessing care for People Living with HIV for Primary Health Care, Recife, Pernambuco, Brazil, 2022 ^1^PrEP - Pre-Exposure Prophylaxis; ^2^PEP - Post-Exposure Prophylaxis; ^3^ARV - Antirretroviral; ^4^VL - Viral Load; ^5^STI - Sexually Transmitted Infection; ^6^ART - Antiretroviral Therapy; ^7^MDS - Medication Dispensing System; ^8^SAS - Specialized Assistance Service

To develop the instrument “Evaluation of the decentralization process of care for People Living with HIV in Primary Health Care”, the logical model presented in Figure 1 was elaborated, based on the triad Structure - Process - Outcomes proposed by Avedis Donabedian^(15)^. Based on the logical model, 68 items distributed in 8 factors were initially identified and proposed for the evaluation.

In the stage of content validation by expert judges, of the 9 expert judges, 6 (66.7%) were female and 3 (33.3%) were male. The age of the judges ranged from 27 to 36 years, with a mean age of 30.78 years (SD = 2.906). The time working in the context of STI/HIV/Aids ranged from 2 to 12 years, with a mean time of 4.22 years (SD=3.492).

All expert judges have a degree in nursing and a specialization degree, 5 (55.6%) with l*ato sensu* specialization in infectology, 3 (33.3%) in public health and 1 (11.1%) in public health. Five (55.6%) of the judges had stricto sensu specialization, in the areas of Biotechnology, Nursing, Public Health, Health Surveillance and Promotion and Health Surveillance.

The expert judges suggested changing the factor physical resources for physical, material and human resources and combining the factors surveillance and notification and prevention in health into a single factor called health surveillance and prevention, totaling 7 factors.

After the judges’ analysis, three items were excluded: (1) Nursing consultation is carried out for HIV positive cases; (2) Medical consultation is carried out for HIV positive cases. Both items obtained the lowest percentage of agreement among the expert judges, 22.2%; and (3) The female condom is well accepted by the health center population, totaling 65 items.

The Kappa index, used to verify the level of agreement between the expert judges, was lower than that recommended in the literature^(24-25)^, which sets a value of 0.75 to indicate an excellent level of agreement. All expert judges presented values lower than this, which pointes to the need for a new data collection, selecting a technical sample with greater practical knowledge (Table 1).

**Table 1:** Kappa index of agreement between the expert judges participating in the content and face validation stage of the instrument for evaluating the decentralization process of care for People Living with HIV in Primary Health Care, Recife, Pernambuco, Brazil, 2022

	Judge 1	Judge 2	Judge 3	Judge 4	Judge 5	Judge 6	Judge 7	Judge 8
**Judge 1**	1							
**Judge 2**	0,432	1						
**Judge 3**	0,454	0,479	1					
**Judge 4**	0,595	0,464	0,688	1				
**Judge 5**	0,427	0,429	0,522	0,577	1			
**Judge 6**	0,455	0,461	0,510	0,518	0,513	1		
**Judge 7**	0,515	0,420	0,482	0,598	0,633	0,465	1	
**Judge 8**	0,481	0,434	0,576	0,697	0,522	0,452	0,509	**1**
**Judge 9**	0,528	0,541	0,563	0,642	0,962	0,568	0,516	**0,542**

A total of 3 technical reviewers participated in the stage of content validation by technical reviewers, all of them female, with a mean age of 47 years old (SD = 13.527) and age ranging between 33 and 60 years old. The mean time of experience was 17 years (SD=10.44), ranging from 5 to 24 years. The Kappa Index showed an agreement of 0.75 between judges 1 and 2; of 0.78 between judges 1 and 3; and of 0.75 between judges 2 and 3.

After the analysis by the technical reviewers, two items were excluded: (1) Patients diagnosed with HIV are separated into symptomatic and asymptomatic and (2) Does the Family Health Strategy offers rapid oral fluid testing for HIV, syphilis and viral hepatitis?, as there was no consensus on the factors answered by the technical reviewers. Therefore, these 2 items were excluded, leaving 63 items divided in 6 factors, namely: 1) Physical, material and human resources (items 1 to 10), 2) Health surveillance and prevention (items 11 to 22), 3) Diagnostic and routine exams (items 23 to 37), 4) Continuing education (items 38 to 41), 5) Education in health (items 42 to 52) and 6) Health Care Network (items 53 to 62).

Item 63 “Does the Family Health Strategy provide antiretroviral drugs?” was the only item that reached consensus on the Treatment factor, suggesting that this factor should not be considered. In this perspective, item 63 should be maintained for further analyses, but in the context of content validation, it should not be kept, as a factor must have at least 3 items^(26)^. Therefore, we proceeded to the semantic validation.

A sample of 55 students aged between 19 and 38 years old (Mean age = 22.45 and SD = 2.860) was used for the semantic validation. The sample consisted of 34 (61.8%) undergraduate nursing students and 21 (38.2%) medical students. Among the participants, 30.9% (17) were in the 6th period, with a mean of 5.82 and SD of 1.857. It was found that > 87.3% of the students selected the option “I understand it completely” for all items of the assessment instrument.

Considering the investigation mentioned above, it is possible to consider that the instrument has content validity, as recommended by the AERA, APA and NCME institutions^(23)^.

## DISCUSSION

The proposal of decentralizing the care for PLHIV in PHC resulted from the need to ensure treatment and have good responses to ARVs^(27-29)^. To achieve the desired results, PLHIV must be engaged in health care, and have continuous access to treatment^(30)^. Thus, care for PLHIV in PHC can enable better management of the transmission of the disease, since it allows the implementation of two fundamental strategies to control the transmission of HIV infection, increased testing and antiretroviral (ARV) coverage^(4,12)^.

Successful experiences with this model of care have been carried out in other countries, such as South Africa. In a study carried out in Zimbabwe, results showed 87.4% of PLHIV were aware of their serological status, 95.3% were on treatment for HIV and 83.2% were virologically suppressed, almost reaching the 95-95-95 goal proposed for the control of HIV infection in the world^(6-7)^.

The decentralization of HIV care in PHC is already a reality in low- and middle-income countries, such as Sub-Saharan Africa, where around 54% of the population lives with HIV. This process aims to facilitate treatment, increasing adherence to ARVs and viral suppression, and reducing possible barriers to care and work overload in outpatient services^(31)^.

Previously focused on specialized services, this new health arrangement characterizes the PHC as a gateway and coordinator of care and Specialized Assistance Services (SAS) as matrix services, with health care being shared between the two levels^(32)^. Recommendations about the follow-up of PLHIV in PHC suggest the creation of a welcoming, unique environment, centered on the patient and free from stigma^(30)^.

Positive aspects include the increase in screening tests and coverage of ARVs and reduction in distances traveled and costs. The negative aspects of decentralization that stand out include: shortage of employees, increased work demand, lack of professional experience, lack of training and support from matrix services, fear and discomfort of patients for being followed up close to their homes^(4,12)^.

In this perspective, the situational diagnosis is important for the evaluation of the process of decentralization of care for PLHIV in PHC. This assessment may be possible through the use of validated instruments. Therefore, the construction of validated instruments is of great importance to reach the objectives of evaluating specific phenomena in different areas. It is worth noting that the evaluation instruments must be reliable and credible to allow assessments that can contribute to strategic decision-making and improvements in health care processes^(33)^.

In this study, with the documentary research, it was possible to create a logical model for the construction of an instrument to evaluate the care provided to PLHIV in PHC, based on the triad Structure-Process-Outcomes. Logical evaluation models are tools that constitute a graphic representation of the theoretical framework of the recommendations for practice. Thus, it was possible to prepare a proposal for the instrument to be validated^(23)^.

Content validation is fundamental in the validity process, as it allows verifying the relevance and representativeness of the proposed items. The validation process by expert judges is the judgment carried out by a group of specialists with experience in the thematic area of the instrument. This process aims to improve the proposed content, making it more accurate, reliable, and valid in what it aims to analyze^(34)^.

Therefore, the results of the study regarding the content validation by expert judges suggested the exclusion of 3 items, of which 2 (23 and 24) addressed nursing and medical consultations for HIV positive patients and obtained the lowest percentage of agreement at this stage. Item 40 was also excluded, as the judges considered that this item evaluated patients’ acceptance/opinion of the female condom. Even though the Kappa coefficient values were below 0.75, requiring a new data collection with technical reviewers, it can be noted that the expert judges made important observations for the improvement of the instrument and for the discussions on the theme.

Some of the points highlighted were the need for a better structure in PHC for the decentralization of this care; the difficulty in diagnosing and monitoring the key population, especially drug users; the importance of the notification of STIs for the planning of preventive actions, considering that there are under-reporting issues, especially in the general population, as testing and notification of pregnant women are prioritized.

Other issues were highlighted, such as: monitoring only in SAS, as they have specific tests, such as CD4 and viral load tests (VL), availability of ARVs and of a multidisciplinary team, with the exception of pregnant women and co-infected children, who are usually followed up in the two levels of care; the application of the rapid test only by nurses, which has been causing work overload, prioritization of specific population, such as pregnant women, and referral of the spontaneous demand to the Testing and Counseling Centers (CTA), as other professionals in PHC refuse to apply the test.

It is evident that the adoption of control measures for HIV is more effective with pregnant women. However, it is necessary to have more effective control measures regarding the application of the rapid test for the spontaneous demand, the sexual partners of those diagnosed with HIV, and women with signs of STIs, welcoming all users who seek the service and enabling prevention, promotion and diagnosis actions in a timely manner^(15,35-36)^.

A national study carried out with nurses who worked in PHC revealed the existence of barriers related to the flow of the healthcare system, the precarious and inadequate physical condition of the health centers, and shortage of materials and supplies. Also, there was a lack of human resources, leading to work overload and lack of training^(11)^. It should be noted that, many times, nurses are the only professionals who get the training and who apply the rapid test, which can lead to increased work demand and interruption of this activity in the service, impairing care^(2,11)^.

The content validation by technical reviewers showed an increase in the level of agreement and a Kappa coefficient higher than that recommended. At this stage, only few observations were made, and the exclusion of only 2 items (5 and 27) was recommended. In item 5, each reviewer suggested a factor, namely: health surveillance, treatment, and Health Care Networks, which justified the exclusion. Item 27 was recommended for exclusion because, in the state of Pernambuco, the rapid oral fluid test is performed only for HIV and offered only by NGOs, which would make it difficult for the target population to respond, according to the reviewers.

As for the semantic validation, the instrument was proofread in Portuguese before proceeding to this stage. The choice of medical and nursing students was due to the need to apply the instrument to a population in a lower level of ability when compared to the target population^(36)^.

This validation step is extremely important, as it reflects the assessment of those who will use the instrument. It should be noted that evaluation instruments must have a cohesive and organized structure, with adequate and sufficient language for their understanding. A logical sequence must be followed, with attention to what is being evaluated^(15,33,37-38)^.

In this perspective, the construction of an instrument to evaluate the process of care for PLHIV in PHC will help in the evaluation of the functioning of the healthcare system for PLHIV, as these patients are often sent on a journey within the system to solve their problems. The (re)structuring of PHC for the care of PLHIV can provide the continuity of care, promote a bond between professionals and patient and enhance the actions aimed at health education, promotion, prevention, diagnosis and treatment of HIV/AIDS, allowing a better management of HIV infection^(4,10)^.

### Limitations of the study

The process of decentralization of the care for PLHIV in PHC in Brazil is at different stages of implementation. This aspect may have impaired the evaluation by the expert judges, as the items of the instrument address issues that would be more clear for professionals who work in services where the process is fully implemented. Thus, the research participants may have answered items not yet experienced in the assistance to PLHIV in PHC.

### Contributions to the area

It is expected that the use of this instrument will enable the implementation of actions aimed at developing the process of decentralization of care for PLHIV in PHC, as well as the identification of the strengths and weaknesses of the process. In addition, it is hoped that the use of this tool will help managers and health professionals in the structuring of health centers that still have a centralized service or in the improvement of health centers that have already started the decentralization process, aiming to guarantee the success of this process and to provide comprehensive care for PLHIV.

## CONCLUSIONS

The development of the logical model supported the construction of the instrument entitled “Evaluation of the process of decentralization of care for People Living with HIV in Primary Health Care”, allowing the identification of the actions aimed at preventing HIV/STI infection and the components to be addressed in primary care in order to promote accessibility and provide quality care for these users.

The instrument was validated for its content, in accordance with the recommendations of the institutions AERA, APA and NCME. The instrument consists of 63 items, divided into 6 factors, namely: Physical, material and human resources; Health surveillance and prevention; Diagnostic and routine tests; Continuing Education; Health education and Health Care Network. Item 63 was kept in the instrument; however, it was the only one that reached consensus on the Treatment factor, suggesting that this factor should not be considered and should be evaluated in new analyses. It is recommended to continue the study on the validation of the instrument’s construct.
